# Community and Research Perspectives on Cancer Disparities in Wisconsin

**DOI:** 10.5888/pcd17.200183

**Published:** 2020-10-08

**Authors:** Jessica Olson, Tobi Cawthra, Kirsten Beyer, David Frazer, Lyle Ignace, Cheryl Maurana, Sandra Millon-Underwood, Laura Pinsoneault, Jose Salazar, Alonzo Walker, Carol Williams, Melinda Stolley

**Affiliations:** 1 Medical College of Wisconsin, Milwaukee, Wisconsin; 2 University of Wisconsin-Madison, Madison, Wisconsin; 3 Gerald L. Ignace Indian Health Center, Milwaukee, Wisconsin; 4 University of Wisconsin-Milwaukee, Milwaukee, Wisconsin; 5 Evaluation Plus, LLC, Milwaukee, WI; 6 Sixteenth Street Community Health Centers, Milwaukee Wisconsin

## Abstract

**Introduction:**

Significant disparities are apparent in geographic areas and among racial/ethnic minority groups in Wisconsin. Cancer disparities are complex and multifactorial and require collaborative, multilevel efforts to reduce their impact. Our objective was to understand cancer disparities and identify opportunities to collaborate across community and research sectors to address them.

**Methods:**

From May 2017 through October 2018, we assembled groups of community members and researchers and conducted 10 listening sessions and 29 interviews with a total of 205 participants from diverse backgrounds. Listening sessions and interviews were scheduled on the basis of participant preference and consisted of a brief review of maps illustrating the breast and lung cancer burden across Wisconsin, and a semistructured set of questions regarding causes, solutions, and opportunities. Interviews followed the same structure as listening sessions, but were conducted between a facilitator and 1 or 2 individuals. Major themes were summarized from all sessions and coded. We used the Model for Analysis of Population Health and Health Disparities to identify areas for collaboration and to highlight differences in emphasis between community participants and researchers.

**Results:**

Participants identified the need to address individual behavioral risks and medical mistrust and to build equitable multilevel partnerships. Communities provided insights on the impact of environment and location on cancer disparities. Researchers shared thoughts about societal poverty and policy issues, biologic responses, genetic predisposition, and the mechanistic influence of lifestyle factors on cancer incidence and mortality.

**Conclusion:**

Listening sessions and interviews provided insight into contributors to cancer disparities, barriers to improving outcomes, and opportunities to improve health. The unique perspectives of each group underscored the need for multisector teams to tackle the complex issue of cancer disparities.

**SUMMARY**

**What is already known about this subject?**

Listening sessions and interviews with community and research groups provided unique insight into factors that contribute to cancer disparities, barriers to improving outcomes, and opportunities to improve health.

**What is added by this report?**

Analyzing data through The Model for Analysis of Population Health and Health Disparities contributed to our understanding of how different groups understand factors associated with disparities and where opportunities for meaningful collaboration exist.

**What are the implications for public health practice?**

The model allowed us to more fully understand the importance of seeking solutions to cancer disparities through a multisector approach rooted in the specific needs of communities.

## Introduction

Cancer incidence and mortality in the United States have decreased overall in recent years, but not equally across all populations. Disparities may be related to race, ethnicity, socioeconomic status, and geographic location, and their underlying causes are complex and multifactorial (1–3). An interplay of biology, individual behavior, socioeconomic status, social conditions, social norms, and environment contribute to disparities in cancer incidence, late-stage diagnosis, and mortality (4–5). In Wisconsin, where cancer is a leading cause of death, significant disparities are apparent in geographic areas and among racial/ethnic minority groups (6–9). Wisconsin has the nation’s second largest Black–White disparity in lung cancer mortality, and the Milwaukee metropolitan area has the largest Black–White disparity in lung cancer mortality among metropolitan areas nationwide (rate ratio = 1.635). Additionally, Wisconsin has the nation’s third largest Black–White disparity in female breast cancer mortality (rate ratio = 1.600) (6,9).

Recognizing the impact lung cancer and female breast cancer have in Wisconsin, the Advancing a Healthier Wisconsin Endowment committed a substantial investment to reduce breast and lung cancer disparities (10). The endowment sought an innovative solution that leveraged the strengths of community-based organizations, population health, and basic science. As a first step, the endowment convened a design team of 10 representatives from research and community settings. The team’s objective was to engage people from different disciplines and communities with varied perspectives on the causes of breast and lung cancer disparities and to inform effective strategies to collaborate across these sectors. To achieve this, the design team conducted statewide focus groups with diverse participants. Team members recommended calling the groups “listening sessions” because facilitators were there to listen, not examine as in a focus group. We describe the listening-session approach and key findings from the sessions.

## Methods

### Recruitment

**Figure 1:**
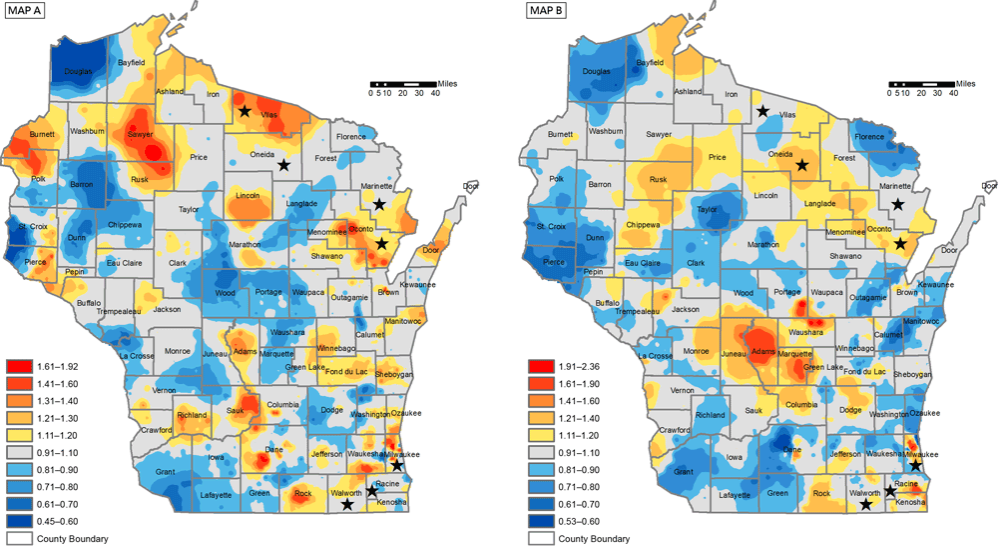
Female breast cancer mortality rate (Map A) and lung cancer mortality rate (Map B), Wisconsin, 2008–2013. The female breast cancer mortality rate is indirectly age standardized and smoothed using adaptive spatial filtering. The lung cancer mortality rate is indirectly age–sex standardized and smoothed using adaptive spatial filtering. A grid of points is used to estimate mortality rates continuously across the map, based on the 20 closest breast cancer deaths and the 40 closest lung cancer deaths. Red areas indicate higher rates than expected and blue areas indicate lower rates than expected, compared with the regional rate. Areas without color indicate rates close to the regional rate. Data source: State Vital Records Office, Wisconsin Department of Health Services 2008-2013 (12). Reprinted with permission of Yuhong Zhou, PhD, and Kirsten Beyer, PhD, MPH, MS, Medical College of Wisconsin.

The design team (authors J.O., T.C., K.B., D.F., L.I., S.M., L.P., J.S., A.W., C.W., M.S.) met regularly from March 2017 to October 2018 and used publicly available maps to identify areas of Wisconsin where lung and female breast cancer rates were higher than expected and where rates of the 2 cancers differed from each other (Figure 1) (11). Nine counties of interest were identified on the basis of apparent disparities in breast and lung cancer incidence and mortality. We contacted public health directors from each county department of health by email to explore their interest in organizing listening sessions and interviews. We sent a follow-up email, followed by a telephone call, to directors who did not respond to the initial email. Of the 9 counties, 7 directors expressed interest, and their counties were included: Marinette, Milwaukee, Oconto, Racine, Vilas, Oneida, and Walworth. The county communities were diverse in their populations’ racial/ethnic make-up and other socioeconomic indicators (Table 1).

**Table 1:** Demographic Characteristics, Wisconsin and 7 Participating Counties, Community and Research Perspectives on Cancer Disparities, May 2017–October 2018

Characteristic	Wisconsin	County						
		Marinette	Milwaukee	Oconto	Oneida	Racine	Vilas	Walworth
**Population**	5,813,434	40,434	948,207	37,830	35,470	196,584	21,938	103,718
**Median household income, $**	59,209	47,497	48,742	57,105	54,198	59,749	44,285	61,106
**Poverty, %**	11.0	12.0	19.1	9.2	9.4	12.6	10.9	10.1
**Uninsured aged <65 y, %**	6.5	6.2	8.1	6.3	6.3	6.2	10.2	8.5
**Race/ethnicity, %**								
White	81.1	95.1	51.0	94.8	94.7	71.7	84.8	85.3
Black	6.7	0.6	27.2	0.4	0.7	12.0	0.4	1.2
American Indian	1.2	0.8	1.0	1.5	1.2	0.7	11.1	1.1
Hispanic/Latino	6.9	1.9	15.4	1.8	1.6	13.4	2.8	11.2
**Female breast cancer 2012–2016**								
Age adjusted incidence rate per 100,000^a^	68.0	62.0	74.6	63.4	78.7	69.7	74.0	68.6
Late-stage diagnosis, % of total cases	32.5	37.2	35.6	36.9	37.3	34.4	27.5	32.2
Age adjusted mortality rate per 100,000^a^	10.7	9.8	11.9	11.7	9.8	10.4	10.7	11.0
**Lung cancer 2012–2016**								
Age adjusted incidence rate per 100,000^a^	59.8	69.8	69.1	64.6	74.6	68.7	72.0	60.0
Late-stage diagnosis, % of total cases	74.3	78.7	77.0	86.0	87.6	74.5	84.3	77.0
Age adjusted mortality rate per 100,000^a^	41.0	48.9	46.6	46.1	48.3	44.9	41.1	45.6

a Age adjusted to 2000 US standard population.

A total of 205 people participated in either listening sessions or interviews from May 2017 through October 2018. To represent the biomedical science groups (bench, clinical, and population health researchers), we invited 50 researchers from Wisconsin and 26 researchers at a national conference. Forty-seven Wisconsin researchers and 20 national researchers accepted. All participants in the biomedical science groups had expertise in cancer and/or disparities research. For this group, 5 sessions were held with a maximum of 11 participants each. In community groups, public health directors who expressed interest in hosting listening sessions invited members of their community that they believed would have insight on cancer incidence in their community. Community participants were leaders from community and nonprofit organizations, community health workers, nonaffiliated community members, directors of federally qualified health centers and free clinics, and public health professionals. We conducted 5 listening sessions (participant number determined by public health director) and 29 interviews (1–2 participants per session) with community groups. We also conducted a listening session at a statewide meeting of Wisconsin’s Centers for Disease Control and Prevention’s National Breast and Cervical Cancer Early Detection Program. For all participants, participating in a listening session or interview was determined by the participant’s preference and availability.

### Listening sessions and interviews

Our multidisciplinary team of community members and researchers conducted 10 listening sessions and 29 interviews with a total of 205 individuals from diverse backgrounds. We developed a format for successful engagement across diverse groups of communities and researchers (Table 2). All listening sessions and interviews were conducted by design team members (authors T.C., J.O., M.S., K.B., C.W.) or trained facilitators with cancer disparities knowledge, public health expertise, and qualitative data collection experience. Listening sessions and interviews were not audio- or video-recorded because public health officials said that participants would engage more freely if not being recorded. A team member took detailed notes on session content and documented observations related to participant affect or interactions at all interviews and listening sessions. Because sessions were not audio- or video-recorded, documenting body language and behavior added context for qualitative analysis. Following each interview or listening session, the notetaker prepared de-identified summaries, and participants were given the opportunity to review them for completeness and accuracy.

**Table 2:** Listening Session and Interview Questions Asked and Participant (N = 205) Characteristics, Community and Research Perspectives on Cancer Disparities, May 2017–October 2018

Listening Session and Interview Format	Justification
**Characteristic**	
Homogeneous	Create an environment where groups feel comfortable sharing experiences.
Facilitated	Enable open conversation that respects cultural, racial/ethnic, or research identities.
Transparent	Ensure the intentions of data collection are clear, and participants understand their ability to stay informed and continue to give feedback throughout the project.
Valid	Seek feedback from a representative from each community after compilation of data, and make modifications, additions, or redactions before dissemination.
Respectful	Establish at the beginning of each listening session or interview that all opinions are valid, and all participants may finish their thoughts without interruption.
Flexible	Tailor sessions to be responsive to participant needs, including group size, style, language, format, and familiarity with the topic of cancer disparities.
**Question**	**Probe (if needed)**
Research: How would you describe the health of Wisconsin communities? Community: How would you describe the health of your community?	Research: Rank the health of Wisconsin communities and explain. Community: Rank the health of your community and explain. (A = Excellent, F = Terrible)
If money or resources were no issue, what would you do to improve cancer disparities?	Are there assumptions that people make about (your community/research)?
Why do maps of breast and lung cancer incidence and mortality look the way that they do?	Are there things that surprise you or don’t surprise you?
**Listening session and interview results, contributors to cancer disparities**	**Examples**
Biologic contributors	Genetic predisposition
Research needs	Better cancer detection, availability of samples from different populations, funding
Behaviors and comorbidities	Obesity, poverty, alcoholism, smoking, diet, exercise, stress, reproductive factors, breastfeeding, use of hormone replacement therapy
Demographic factors	Health literacy, gender, race/ethnicity, childhood education
Geographic location	Distance to care, location of care, availability of transportation
Environment	Airborne, housing, and workplace exposures, radon, water quality
Social conditions	Social isolation, cultural norms, social support
Institutional barriers	Availability of quality care, patient support, availability of partnerships and funding sources, medical mistrust
Policy issues	Insurance coverage, societal poverty, generic drug availability, adherence to policy

Interviews were scheduled for 60 minutes and listening sessions for 90 minutes. Questions and probes were determined a priori by the design team to capture research and community perspectives on causes and challenges contributing to breast and lung cancer disparities statewide and opportunities to improve health outcomes. To ensure that the verbiage of questions would be understood across community and research populations, the design team tested the applicability of questions across diverse groups with peers and social networks and used their feedback to inform revisions. At listening sessions, the facilitator encouraged participants to openly share their perceptions of their home community, communities statewide, and the environment of cancer research. Participants then examined statewide maps of breast and lung cancer incidence and mortality and discussed whether what they saw in the maps validated or opposed their previous thoughts about community health and cancer disparities. At the end of the listening sessions and interviews, participants were encouraged to ask questions about future directions and were informed of ways to stay connected to the study.

### Data analysis

Two trained researchers (T.C., J.O.) coded summaries and observational notes using ATLAS.ti software (ATLAS.ti Scientific Software Development GmbH). In the first round of coding, researchers used open coding to identify themes, key concepts, ideas, beliefs, or events. Researchers met frequently to compare and modify codes and resolve discrepancies through discussion or consultation with a third reviewer. After completing open coding of themes (Table 2), the themes that emerged strongly aligned with The Model for Analysis of Population Health and Health Disparities, a model that illustrates multilevel contributors to cancer disparities, including individual behavior and risk, context, and population factors (13). A second round of coding was then conducted to help identify thematic similarities and differences between researchers and community members to inform opportunities for collaboration or identify experiential gaps that might require further attention (Table 3). All procedures were reviewed and approved by the Medical College of Wisconsin’s institutional review board.

**Table 3:** Topics Discussed in Listening Sessions and Interviews, Community and Research Perspectives on Cancer Disparities, May 2017–October 2018^a^

Topic of Discussion	Research Participants		Community Participants (n = 158)
	Basic/Clinical (n = 36)	Population Health (n = 11)	
**Biologic and genetic pathways**			
Availability of technology, samples, and models	X		
Genetic predisposition	X	X	X
Mechanisms of protection or damage	X		
**Biologic responses**			
Alcohol, obesity, and stress	X	X	X
**Individual risk factors**			
Medical mistrust, delay to diagnosis, completion, adherence to care	X	X	X
Reproductive/gynecologic factors	X	X	
Individual diet, alcohol, tobacco, and illegal drug use	X	X	X
**Individual demographics**			
Access to care		X	X
Childhood and community education		X	X
Cultural and acculturation		X	X
Gender and race	X	X	X
Employment and socioeconomic status		X	X
**Physical context**			
Environment (agriculture, home, community, workplace exposures)	X	X	X
Location (urban, rural, green, isolated)	X	X	X
**Social relationships**			
Acceptability of alcohol consumption and smoking	X	X	X
Social factors (support, isolation, pride, self-efficacy)		X	X
**Social context**			
Effectiveness of partnerships	X	X	X
Social capital	X	X	X
**Institutional context**			
Adequate patient support, care, and physician training	X	X	X
Capacity for multidisciplinary work	X	X	
Guideline concordant care, hospital volume, cancer detection		X	
Need for champions and funding opportunities	X		X
**Social conditions and policy **			
Environment, housing, and insurance-based policy		X	X
Insurance issues		X	X
Social inequality and societal poverty	X	X	X

a X indicates that the topic was discussed in the basic/clinical research, population health research, and/or community groups.

## Results

### Interviews and listening session participants

**Figure 2:**
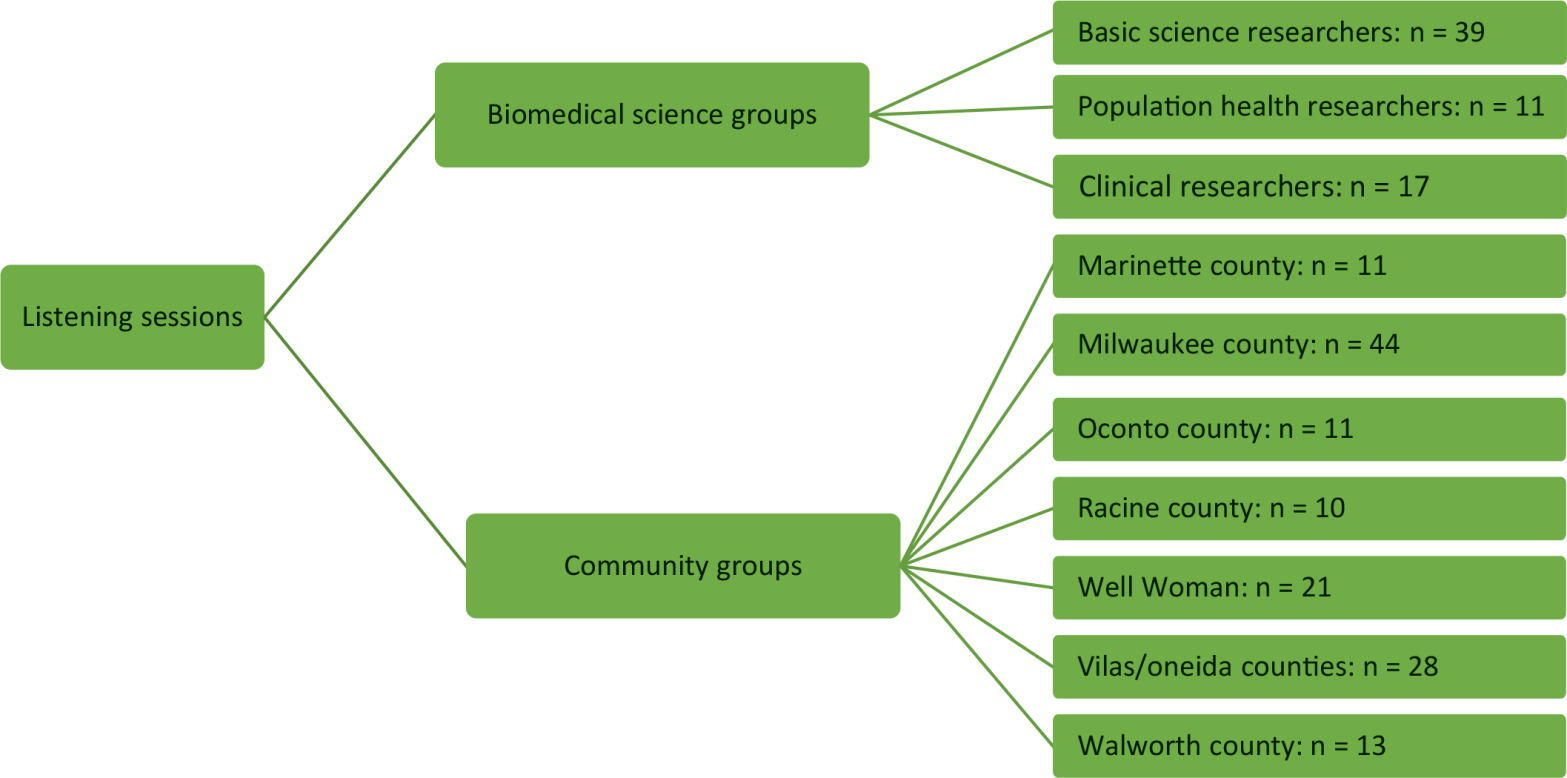
Composition of listening sessions and interviews. A total of 205 participants answered semistructured questions about communities and cancer disparities in Wisconsin. Sixty-seven participants represented basic, population health, and clinical research, and 138 participants represented community perspectives. We also conducted a listening session at a coordinators meeting of Well Woman, the Wisconsin’s Centers for Disease Control and Prevention’s National Breast and Cervical Cancer Early Detection Program.

Listening session and interview participants totaled 205. Twenty-nine interviews were conducted across Wisconsin counties (Marinette, 10 interviews; Oconto, 6; Racine, 7; and Walworth, 6) and consisted of either 1 or 2 participants per interview for a total of 35 people interviewed and 170 participants in listening sessions (Figure 2).

Open coding revealed a broad range of contributors to cancer disparities: biologic contributors, research needs, behaviors and comorbidities, demographic factors, geographic location, environment, social conditions, institutional barriers, and policy issues (Table 2).

**Biologic contributors**. All sessions acknowledged genetic predisposition for cancer. Modifiable risk factors were believed to be the predominant contributors to cancer disparities, but researchers recognized that some communities were possibly more likely than others to experience geographic disparities through rural isolation and small community size and therefore inherit cancer-causing genes disproportionately.

**Research needs**. Researchers acknowledged difficulties in recruiting diverse populations for sample collection and clinical trials. One researcher said she had diverse racial and ethnic participation when she began recruitment for a clinical trial, but by the end, “all of the non-White participants had dropped out,” and she had no idea why. Another basic scientist said he was “aware of disparities in cancer incidence” within the type of cancer he studied but was unsure about how to incorporate that into his animal-based research.

**Behaviors and comorbidities**. All listening sessions discussed the considerable impact of smoking, stress, diet, and lack of physical activity on rates of cancer incidence and mortality. Researchers also discussed the impact of reproductive factors, such as parity, breastfeeding practices, and the use of hormone replacement therapy on breast cancer. Community participants had specific ideas to improve health outcomes that would address local concerns. For example, in one rural area of Wisconsin with high levels of summer tourism, community members said that walking paths in the area would be used by local residents much more often if the paths actually went places (like the grocery store), instead of in circles (for the tourists). Another community participant said that a great opportunity to conduct an intervention would be at “thresherees,” which are gatherings of local agricultural community members during harvest seasons.

**Demographic factors**. Community health care providers said that many of their current research efforts focused on educating communities and increasing knowledge and awareness of cancer-causing agents. In 5 of the 7 counties visited, health care providers shared that adults in their area were aware that they should eat better, be more active, and either eliminate or reduce tobacco and alcohol consumption, yet had little interest in modifying behavior.

**Geographic location**. Both community participants and researchers discussed the influence of distance and travel time on health care, but these were not the sole concerns related to access. In 2 separate listening sessions, participants said that they would “have to be dying” to seek care at their local health care facility and would prefer to drive an hour or more to larger cities for what they trusted to be better quality care. In urban settings, mistrust stemmed from experiences and beliefs that care would be delivered differently because of the race, ethnicity, or socioeconomic status of the patient. Researchers and public health experts discussed this mistrust but did not acknowledge its nuances in different demographic groups.

**Environment**. Community participants expressed concerns about airborne, housing, and workplace exposures to harsh chemicals and environmental pollutants, which differed by region. In northern Wisconsin, industrial chemicals found in paper mills and mining were mentioned, and in agricultural areas throughout the state, exposure to pesticides and herbicides were referenced as concerns. Participants from urban areas expressed more concern about pollution and quality of housing. Researchers acknowledged the impact of the environment on health and were knowledgeable about the high levels of radon in certain Wisconsin communities, but did not focus discussions on any other community-specific exposures.

**Social conditions**. Population health researchers and community participants shared that a significant disparity between communities exists in the way that tobacco and alcohol are promoted. Sale of tobacco and alcohol is promoted in areas where racial/ethnic and sex and gender minority groups reside, whereas health care, healthy foods, and healthy behaviors are promoted more in suburban, affluent areas with predominantly White populations. In rural communities, participants said, “everybody smokes” and “everybody drinks.” A public health professional said in an interview that tobacco use was so prevalent that when young women become pregnant, they merely switched from cigarettes to chewing tobacco for the duration of their pregnancy. Participants from communities across Wisconsin said that alcohol is expected at all social gatherings.

**Institutional barriers**. All participants acknowledged the institutional challenges to reducing cancer disparities. Researchers cited challenges in obtaining funding, building new partnerships, and then sustaining connections when funding runs out. In communities, institutional barriers were centered around the limited time or resources to form new partnerships and launch programs and the shortage of physicians in an area. A rural nurse practitioner shared that many community members were unwilling (because of perceptions) or unable (for insurance reasons) to receive care from nurse practitioners or health professionals with nonterminal degrees.

**Policy issues**. Listening sessions revealed issues with insurance and generic drug costs, societal poverty, and challenges in banning carcinogenic substances. In multiple listening sessions in northern Wisconsin, community participants said that despite the presence of a statewide indoor smoking ban, smoking was still prevalent in taverns, restaurants, and other public places. Community groups discussed agricultural pesticide use and said that determination of which chemicals are allowed is based on their cost and farmers’ preference without consideration for the health of community members.

By using the Model for Population Health and Health Disparities as a framework to compare research and community perspectives, we were able to compare areas of emphasis between groups. The model served as a powerful tool to identify areas with shared knowledge for future multisector collaboration (Table 2) and areas where more education was likely needed (Table 3).

**Areas with shared knowledge**. Areas with shared knowledge indicated topics with potential for rapid, multisector collaboration. For example, all participant groups discussed the contribution of individual risk factors to cancer disparities but had different expertise and interests in the topics discussed. Alcohol consumption was identified as a contributing factor to cancer across groups, and basic science researchers focused on understanding cellular and molecular mechanisms and discussed work being conducted by local experts that could be focused on state-level issues (14). Population health researchers focused on frameworks that drive lifestyle choices, such as the Transtheoretical Model, and successful interventions to improve health outcomes (15,16). Community participants focused on the social and cultural norms specific to their area.

**Areas with differences in emphasis between participant groups**. Areas where emphasis differed between groups showed that more education would likely be needed to create multisector teams. For example, basic science researchers focused heavily on the mechanisms of DNA and cellular damage and protective factors that need to be better understood. Only researchers mentioned how reproductive and gynecologic factors such as breastfeeding practices, parity (having borne children), and the use of hormone replacement therapy were factors in breast cancer incidence. Community participants had unique insights regarding the physical context of cancer disparities, that is, how the environment and location affect health outcomes. They went into detail about specific agricultural, industrial, workplace, and home exposures that may affect health. For example, in Northern Wisconsin, heavy snowfall can block roads and prevent trucks from delivering propane to heat homes throughout winter and into April and May. To compensate for this, some residents switch to burning wood as a heat source. Wood smoke is a source of benzene, defined as a carcinogen by the International Agency of Research on Cancer. However, limited research has examined the correlation between home heating with open fires or closed burners and cancer incidence (17). Although researchers discussed how social context in general contributes to cancer disparities, community participants had extensive knowledge about the complex, community-specific interplay of social relationships, social conditions, and policy.

### Use of maps to stimulate discussion

We found use of maps to be a critical factor in our investigation. Although both community and research groups tended to focus on the maps or the specific geographic elements where disparities were high, the maps were useful in helping participants go beyond their initial thoughts on factors influencing disparities. As a result of sharing maps, researchers who were previously unaware of cancer disparities were eager to learn more and share access to research equipment (such as next generation imaging and sequencing technology), collaborators, and expertise. Community participants in urban areas were largely aware of cancer disparities, but seeing the warm or hot colors on the maps illustrating the additional burden in their region resulted in comments of interest, dismay, confusion, and commitment (“we need to do something about this”). In rural communities, where initial conversations focused on the fresh air, outdoor activities, and environment that are healthier than that of urban settings, participants were surprised by the maps. Seeing the warm or hot colors on their rural regions on the map indicating high cancer incidence and mortality shifted the discussion to possible causes, such as industrial and agricultural exposures, cultural norms, and health care quality.

## Discussion

The US Department of Health and Human Services called for the elimination of health disparities and achievement of health equity in Healthy People 2020 (4). Our statewide listening sessions and interviews with community members and researchers uncovered multisector factors that contribute to disparities. Previous studies used this listening session approach to uncover barriers between community and science that need to be addressed to reduce health disparities, such as cultural humility and skepticism and mistrust about research (18,19). In our study, we sought to understand the differences in emphasis that diverse participants in research and community settings would place on causes of cancer disparities. When we used disparities-based frameworks in different settings (such as research vs community settings), focus on aspects of the Model for Analysis of Population Health and Health Disparities (13) shifted. This is likely a major reason that disparities are difficult to address. It is challenging for participants to draw their focus from what is most salient to them and examine broader perspectives. We found that each group offered a unique perspective based on their expertise and experience and acknowledged that other sectors also needed to make a significant impact to reduce cancer disparities. Across communities, there was a shared desire to improve health outcomes, and multiple suggestions were offered as first steps. All groups wanted to reduce disparities and improve health outcomes and identified the following 3 key issues to address.

**Major contributors to cancer disparities are complex and vary between regions and communities.** The root causes of cancer disparities are complex and multifactorial. Eliminating cancer disparities statewide requires consideration of the unique factors among communities that underlie disparities. Our statewide listening sessions revealed isolated incidents of environmental contamination, deeply ingrained cultural norms, and institutional barriers that all need to be acknowledged. Nationwide, it is clear that a one-size-fits-all approach across diverse community settings is not sufficient. Previous studies have demonstrated that risk factors contributing to mortality and prognoses differ between races and geographic locations (20,21). The Model for Analysis of Population Health and Health Disparities (13) illustrates the impact of many proximal, distal, and intermediate factors on health. When examining the contributors to health disparities outlined in our listening sessions, factors from each category of this framework emerged (Table 3). Unlike smaller studies that highlighted a central focus for interventions, statewide efforts require interventions that can be tailored to the cultural and geographic needs of the communities affected by cancer disparities (18,19).

**Shared knowledge between researchers and community members is needed**. Researchers and community groups discussed differing priorities regarding cancer disparities. In our listening sessions, researchers expressed a need for more diverse participation in clinical trials and biospecimen donations. This was a recurrent theme across basic, clinical, and population researcher sessions. Aside from issues of medical mistrust, confusion about the importance of clinical trials is prevalent in communities nationwide, and conceptual frameworks have been created to maximize diverse participation in trials (22–24). In our community listening sessions, clinical trials and biospecimen sample donation were not mentioned. Shared understanding, identification, capacity building, and removal of individual and system-level barriers will be required to bridge the gap between community and research priorities (25–28).

**Multisector partnerships are needed to eliminate cancer disparities. **Our study showed that broad understanding and appreciation for local social, cultural, and biological influences on cancer disparities is needed in a multisector team setting to achieve health equity in Wisconsin. Efforts are needed to bridge gaps in communication regarding sample donation and disease model development, which basic science researchers valued more than did population health researchers or community members. Basic scientists, conversely, had limited expertise in how social conditions and policy influence health disparities. Given the community and research perspectives on cancer disparities that we observed, educational approaches or guided facilitation will be required to create collaborative efforts. One opportunity to accomplish this would be through the development of training programs that intentionally bring interested participants from biomedical research (basic and population science) and community settings together to learn about each other’s worlds and to inform research questions that meet community concerns.

One of our most encouraging findings was the acknowledgment across groups of a need for partnerships, improved training, and patient support. Both researchers and community groups acknowledged that funds and time are limited resources; however, they referenced small coalitions and existing partnerships focused on cancer disparities and population health that have had success in outreach programming or grant funding efforts. Although individuals in all sectors expressed willingness to be a part of a larger collaborative group, partnerships between researchers and community generally do not occur organically. These relationships and interactions need to be fostered and facilitated to ensure equity in influence and outcomes. Although a capacity needs to be built to conduct multidisciplinary, cross-cutting work, research and community-based resources, opportunities, and enthusiasm exist to reduce breast and lung cancer incidence and mortality statewide. Ultimately, our study informed effective strategies for multidisciplinary teams to understand cancer disparities and to collaborate across sectors. This approach is recommended for large- or small-scale initiatives to address complex, multifactorial health issues.

Our study had limitations. The design team did not frame its questions around the Model for Population Health and Health Disparities (13). Also, the absence of discussion of an issue did not necessarily mean an absence of understanding or a lack of desire to address an issue at a research or policy level. Listening sessions were approximately 90 minutes long, and in some groups, discussion was extended around some topics, which limited the amount of time for discussion of other topics. Listening sessions were not taped or transcribed; therefore, our analysis relied on the accuracy of notetaking.

## References

[1] American Cancer Society. Cancer facts & figures 2014. Atlanta (GA): American Cancer Society; 2014.

[2] Ward EM , Sherman RL , Henley SJ , Jemal A , Siegel DA , Feuer EJ , Annual report to the nation on the status of cancer, featuring cancer in men and women age 20–49 years. J Natl Cancer Inst 2019;111(12):1279–97. 10.1093/jnci/djz106 31145458 PMC6910179

[3] Kohler BA , Sherman RL , Howlader N , Jemal A , Ryerson AB , Henry KA , Annual report to the nation on the status of cancer, 1975–2011, featuring incidence of breast cancer subtypes by race/ethnicity, poverty, and state. J Natl Cancer Inst 2015;107(6):djv048. 10.1093/jnci/djv048 25825511 PMC4603551

[4] US Department of Health and Human Services. Secretary’s Advisory Committee On Health Promotion and Disease Prevention Objectives For 2020. Phase I report: recommendations for the framework and format of Healthy People 2020. https://www.healthypeople.gov/2020/about/advisory/Reports. Accessed July 30, 2020.

[5] National Cancer Institute. Cancer disparities. 2019. https://www.cancer.gov/about-cancer/understanding/disparities. Accessed April, 17, 2020.

[6] Beyer KMM , Laud PW , Zhou Y , Nattinger AB . Housing discrimination and racial cancer disparities among the 100 largest US metropolitan areas. Cancer 2019;125(21):3818–27. 10.1002/cncr.32358 31287559 PMC6788939

[7] Bemanian A , Cassidy LD , Fraser R , Laud PW , Saeian K , Beyer KMM . Racial disparities of liver cancer mortality in Wisconsin. Cancer Causes Control 2019;30(12):1277–82. 10.1007/s10552-019-01232-9 31531799 PMC6858574

[8] Schrager S . Breast cancer: addressing disparities, improving care. WMJ 2018;117(2):52–3. 30048572

[9] Beyer KM , Zhou Y , Matthews K , Hoormann K , Bemanian A , Laud PW , Breast and colorectal cancer survival disparities in southeastern Wisconsin. WMJ 2016;115(1):17–21. 27057575

[10] Advancing a Healthier Wisconsin Endowment. Advancing a Healthier Wisconsin, Annual Report 2017. https://ahwendowment.org/AHW1/AnnualReports/2016_2017_Annual_Report.pdf. Accessed May, 20, 2020.

[11] Wisconsin Department of Health Services. Vital Records Services. https://www.dhs.wisconsin.gov/vitalrecords/index.htm. Accessed July 30, 2020.

[12] Cancer in Eastern Wisconsin. 2020 https://www.mcw.edu/departments/cancer-center/community-outreach-and-engagement/cancer-in-eastern-wisconsin. Accessed April 16, 2020.

[13] Warnecke RB , Oh A , Breen N , Gehlert S , Paskett E , Tucker KL , Approaching health disparities from a population perspective: the National Institutes of Health Centers for Population Health and Health Disparities. Am J Public Health 2008;98(9):1608–15. 10.2105/AJPH.2006.102525 18633099 PMC2509592

[14] Knight JM , Rizzo JD , Logan BR , Wang T , Arevalo JM , Ma J , Low socioeconomic status, adverse gene expression profiles, and clinical outcomes in hematopoietic stem cell transplant recipients. Clin Cancer Res 2016;22(1):69–78. 10.1158/1078-0432.CCR-15-1344 26286914 PMC4703514

[15] Prochaska JO , DiClemente CC , Norcross JC . In search of how people change. Applications to addictive behaviors. Am Psychol 1992;47(9):1102–14. 10.1037/0003-066X.47.9.1102 1329589

[16] Stolley M , Sheean P , Gerber B , Arroyo C , Schiffer L , Banerjee A , Efficacy of a weight loss intervention for African American breast cancer survivors. J Clin Oncol 2017;35(24):2820–8. 10.1200/JCO.2016.71.9856 28628363 PMC5562172

[17] Bailey HD , de Klerk NH , Fritschi L , Attia J , Daubenton JD , Armstrong BK , ; Aus-ALL Consortium. Refuelling of vehicles, the use of wood burners and the risk of acute lymphoblastic leukaemia in childhood. Paediatr Perinat Epidemiol 2011;25(6):528–39. 10.1111/j.1365-3016.2011.01224.x 21980942

[18] Erves JC , Mayo-Gamble TL , Malin-Fair A , Boyer A , Joosten Y , Vaughn YC , Needs, priorities, and recommendations for engaging underrepresented populations in clinical research: a community perspective. J Community Health 2017;42(3):472–80. 10.1007/s10900-016-0279-2 27812847 PMC5408035

[19] Webb Hooper M , Mitchell C , Marshall VJ , Cheatham C , Austin K , Sanders K , Understanding multilevel factors related to urban community trust in healthcare and research. Int J Environ Res Public Health 2019;16(18):E3280. 10.3390/ijerph16183280 31500126 PMC6765868

[20] Park J , Blackburn BE , Rowe K , Snyder J , Wan Y , Deshmukh V , Rural-metropolitan disparities in ovarian cancer survival: a statewide population-based study. Ann Epidemiol 2018;28(6):377–84. 10.1016/j.annepidem.2018.03.019 29705053 PMC6005362

[21] Rodriguez EA , Tamariz L , Palacio A , Li H , Sussman DA . Racial disparities in the presentation and treatment of colorectal cancer: a statewide cross-sectional study. J Clin Gastroenterol 2018;52(9):817–20. 10.1097/MCG.0000000000000951 29095418

[22] Trantham LC , Carpenter WR , DiMartino LD , White B , Green M , Teal R , Perceptions of cancer clinical research among African American men in North Carolina. J Natl Med Assoc 2015;107(1):33–41. 10.1016/S0027-9684(15)30007-9 26113749 PMC4477827

[23] Barrett NJ , Rodriguez EM , Iachan R , Hyslop T , Ingraham KL , Le GM , Factors associated with biomedical research participation within community-based samples across 3 National Cancer Institute-designated cancer centers. Cancer 2020;126(5):1077–89. 10.1002/cncr.32487 31909824 PMC7021578

[24] Napoles A , Cook E , Ginossar T , Knight KD , Ford ME . Applying a conceptual framework to maximize the participation of diverse populations in cancer clinical trials. Adv Cancer Res 2017;133:77–94. 10.1016/bs.acr.2016.08.004 28052822 PMC5542779

[25] Davis M , Tripathi S , Hughley R , He Q , Bae S , Karanam B , AR negative triple negative or “quadruple negative” breast cancers in African American women have an enriched basal and immune signature. PLoS One 2018;13(6):e0196909. 10.1371/journal.pone.0196909 29912871 PMC6005569

[26] Pérez-Mayoral J , Soto-Salgado M , Shah E , Kittles R , Stern MC , Olivera MI , Association of genetic ancestry with colorectal tumor location in Puerto Rican Latinos. Hum Genomics 2019;13(1):12. 10.1186/s40246-019-0196-4 30786938 PMC6383234

[27] Xiao J , Cohen P , Stern MC , Odedina F , Carpten J , Reams R . Mitochondrial biology and prostate cancer ethnic disparity. Carcinogenesis 2018;39(11):1311–9. 10.1093/carcin/bgy133 30304372 PMC6292412

[28] Reams RR , Kalari KR , Wang H , Odedina FT , Soliman KF , Yates C . Detecting gene–gene interactions in prostate disease in African American men. Infect Agent Cancer 2011;6(Suppl 2):S1. 10.1186/1750-9378-6-S2-S1 21992608 PMC3194179

